# Expression and prognostic characteristics of m^5^C regulators in low‐grade glioma

**DOI:** 10.1111/jcmm.16221

**Published:** 2021-01-05

**Authors:** Xiaozhi Li, Yutong Meng

**Affiliations:** ^1^ Department of Neurosurgery Shengjing Hospital of China Medical University Shenyang China; ^2^ Department of Stomatology Shengjing Hospital of China Medical University Shenyang China

**Keywords:** low‐grade glioma, m^5^C, prognosis, RNA methylation

## Abstract

Glioma is the most common intracranial malignant tumour. A clear diagnosis and molecular targeted therapy are of great significance for improving the survival time and quality of life of patients with low‐grade glioma. 5‐methylcytosine methylation is one of the ways of RNA modification, but there are limited studies on the role of m^5^C methylation of low‐grade glioma. Single‐nucleotide variant, RNA expression matrix and corresponding clinical data of low‐grade glioma came from public database. The single‐nucleotide variant and expression of m^5^C regulators were estimated. A prognostic model based on m^5^C regulators was constructed by Cox regression. Potential functions of these molecules were assessed by gene set enrichment analysis. DNMT3A mutation was the most frequent among the m^5^C regulators in low‐grade glioma. NSUN3, TET2, TRDMT1, ALYREF, DNMT3B, DNMT1, NOP2 and NSUN2 were up‐regulated. One prognostic model was constructed which had a strong predictive power for the overall survival of low‐grade glioma. We studied the expression and prognostic characteristics of m^5^C regulators in low‐grade glioma, supplied biomarkers for the diagnosis and prognosis and provided the foundation for the study of the pathogenesis of low‐grade glioma.

## 1 INTRODUCTION

Glioma is the most common intracranial malignant tumour, and is traditionally classified into low‐grade glioma and high‐grade glioma. With the development of molecular detection and sequencing technology, the distinction of low‐grade and high‐grade glioma has transformed from histology to molecular markers.^1^ With reference to molecular status such as IDH and 1p/19q, the probability of misclassification of glioma based on traditional histology is about 6%‐35%.^2^ In 2016, WHO carried out a molecular classification of glioma, and indicated that molecular characteristics had an important impact on the diagnosis and prognosis of glioma.^3^ The median survival time of patients with low‐grade glioma is about 5‐10 years.^4^ A clear diagnosis and molecular targeted therapy are of great significance for improving the survival time and quality of life of patients with low‐grade glioma.

RNA modification has long been discovered by researchers,^5^ and this reversible change plays a role in regulating gene expression and RNA stability, regulating alternative splicing and other biological functions.^6^, ^7^, ^8^, ^9^ Among them, 5‐methylcytosine (m^5^C) methylation is one of the ways of RNA modification, and this kind of RNA modification exists widely in coding and non‐coding regions in transcriptome.^10^ A variety of regulatory molecules participate in m^5^C methylation process. The methyltransferases including NOP2, NSUN2, NSUN3, NSUN4, NSUN5, NSUN6, NSUN7, DNMT1, TRDMT1, DNMT3A and DNMT3B act as ‘writers’ of m^5^C methylation. In addition, the ‘reader’ of m^5^C methylation (ALYREF) is involved in the stabilization and recognition process of m^5^C methylation.^11^ TET2 is the ‘eraser’ of m^5^C methylation. m^5^C regulates varieties process such as RNA output, RNA stability and translation process.^12^ However, there are limited studies on the role of m^5^C methylation of low‐grade glioma.

This study intends to analyse the expression and prognostic characteristics of m^5^C regulators in low‐grade glioma based on high‐throughput sequencing databases, identify biomarkers and construct a prognostic model, and provide a target for the research and treatment of low‐grade glioma.

## 2 MATERIALS AND METHODS

### 2.1 Acquisition of low‐grade glioma datasets

Single‐nucleotide variant (SNV) data of low‐grade glioma were acquired from the TCGA (https://cancergenome.nih.gov/) database. The RNA expression matrix and clinical data of low‐grade glioma came from the TCGA database (ID: TCGA‐LGG), CGGA database (http://www.cgga.org.cn/, ID: mRNAseq_693 and mRNAseq_325). Meanwhile, the clinical data of low‐grade glioma in TCGA were extracted form cBioportal (http://www.cbioportal.org/) website, including basic information basic information (gender, age, etc), survival data (survival time and survival status) and molecular status (IDH, 1p/19q, TERT mutation, ATRX mutation and MGMT methylation, etc) Genes were annotated by the Ensemble human genome browser (http://asia.ensembl.org/index.html). All data were corrected using ‘limma’ package of R language. Besides, ‘sva’ package was used to merge data of CGGA datasets (mRNAseq_693 and mRNAseq_325).

### 2.2 Construction of a prognostic model based on m^5^C regulators

The TCGA dataset was used as the training cohort to construct a prognostic model, and the CGGA dataset was used as the validation cohort in this study. First, univariate Cox regression was used to identify the m^5^C regulators related to the prognosis of low‐grade glioma. Furthermore, multivariate Cox regression was used to construct a prognostic model and a nomogram of low‐grade glioma based on m^5^C. The risk score was calculated by ∑β_gene n_ × Expression_gene n_. Kaplan‐Meier survival curve was introduced to compare the OS (overall survival) of different risk groups divided by the median risk score. In addition, receiver operating curve (ROC) of 1, 3 and 5 years were used to test the predictive ability of the model for low‐grade glioma.

### 2.3 Gene set enrichment analysis (GSEA)

We estimated the Pearson correlation coefficients between genes with m^5^C regulators to find m^5^C regulator‐related factors. The genes with correlation coefficient > 0.4 (or < −0.4) and *P* < .05 were defined as m^5^C regulator‐related factors, and GSEA was performed based on these factors by ‘clusterProfiler’ package of R language.

### 2.4 Statistical analysis

All statistics and graphics in this study were implemented based on R language (4.0.0). We used the Wilcoxon rank‐sum test rather than the t test to determine whether difference in two groups was statistically significant in this study, because the Wilcoxon test minimizes the influence of outliers compared with t test. Log‐rank test was used to compare different risk groups in the Kaplan‐Meier curve. It was considered statistically different when *P* < .05.

## 3 RESULTS

### 3.1 Mutation characteristics of m^5^C regulators in low‐grade glioma

**FIGURE 1:**
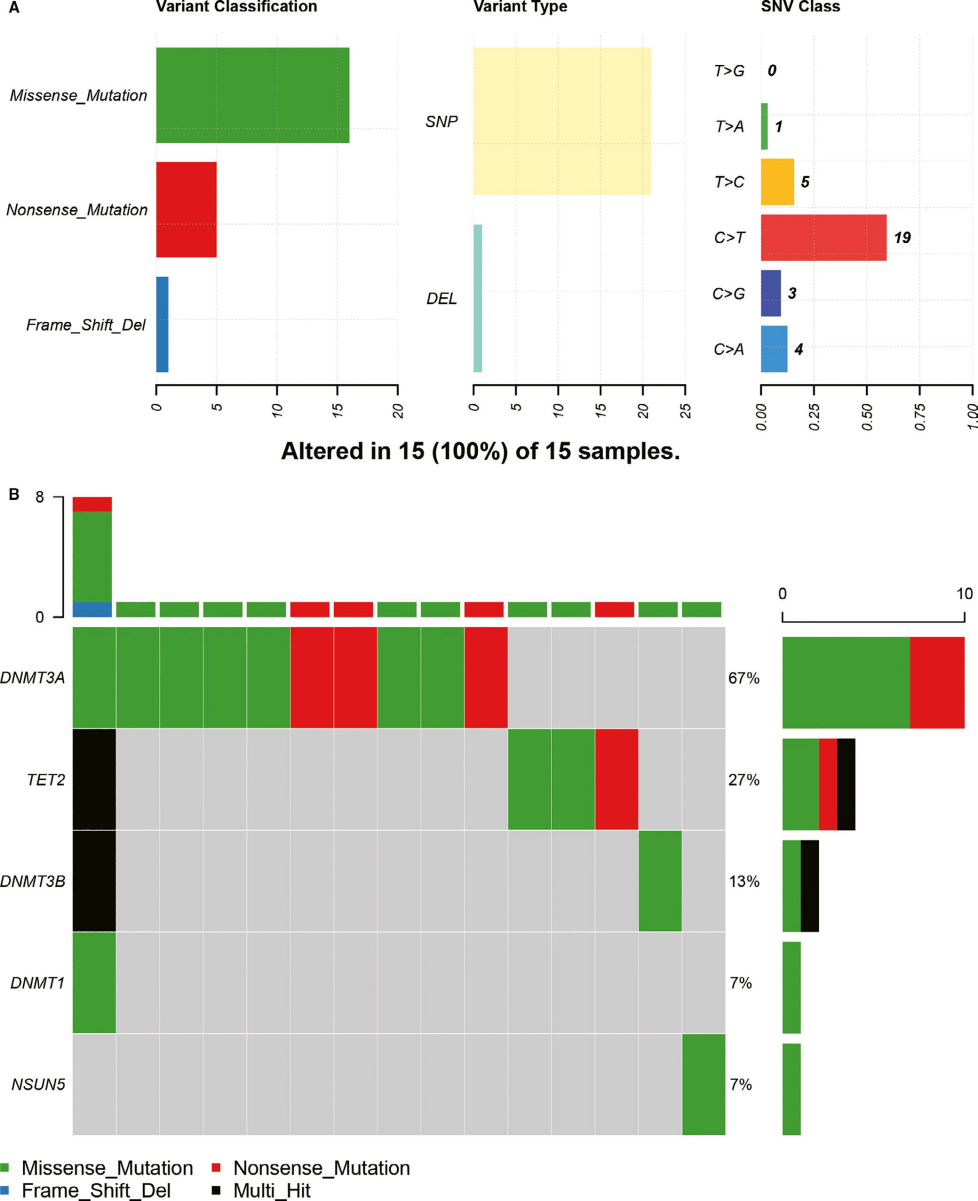
Summary of low‐grade glioma mutation profiles. A, Missense mutation, SNP and C > T were more common in low‐grade glioma mutation. B, Overview of mutation profiles of m^5^C regulators (DNMT3A, TET2, DNMT3B, DNMT1 and NSUN5) in each sample

The ‘maftools’ package of R language was used to analyse the single‐nucleotide variants (SNV) of low‐grade glioma in TCGA dataset. Among all 506 low‐grade glioma mutation samples, there were 15 samples with m^5^C regulator mutations, as shown in Figure 1. The main variant class of m^5^C regulators was missense mutation, followed by nonsense mutation and frame shift deletion. Single‐nucleotide polymorphisms (SNP) occurred more frequently than insertions and deletions (DEL). C > T was the most common SNV in low‐grade glioma. Moreover, the waterfall chart showed the mutation characteristics of each sample. 5 out of 13 m^5^C regulators had SNV mutations, including DNMT3A, TET2, DNMT3B, DNMT1 and NSUN5. Besides, the mutation of DNMT3A was the most frequent.

### 3.2 Differentially expressed m^5^C regulators

**FIGURE 2:**
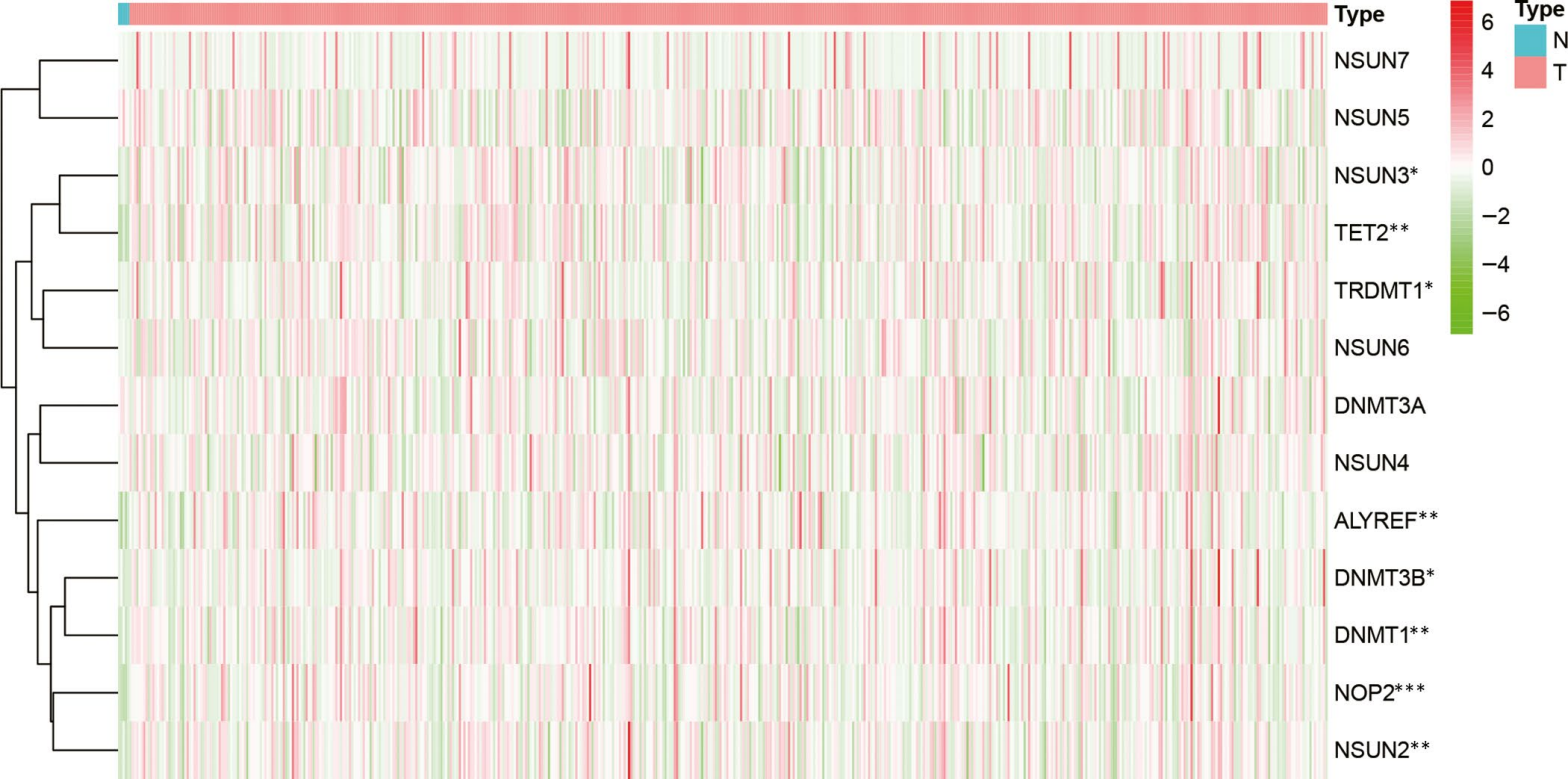
Heatmap of m^5^C regulators in low‐grade glioma. NSUN3, TET2, TRDMT1, ALYREF, DNMT3B, DNMT1, NOP2 and NSUN2 were up‐regulated in low‐grade glioma

**FIGURE 3:**
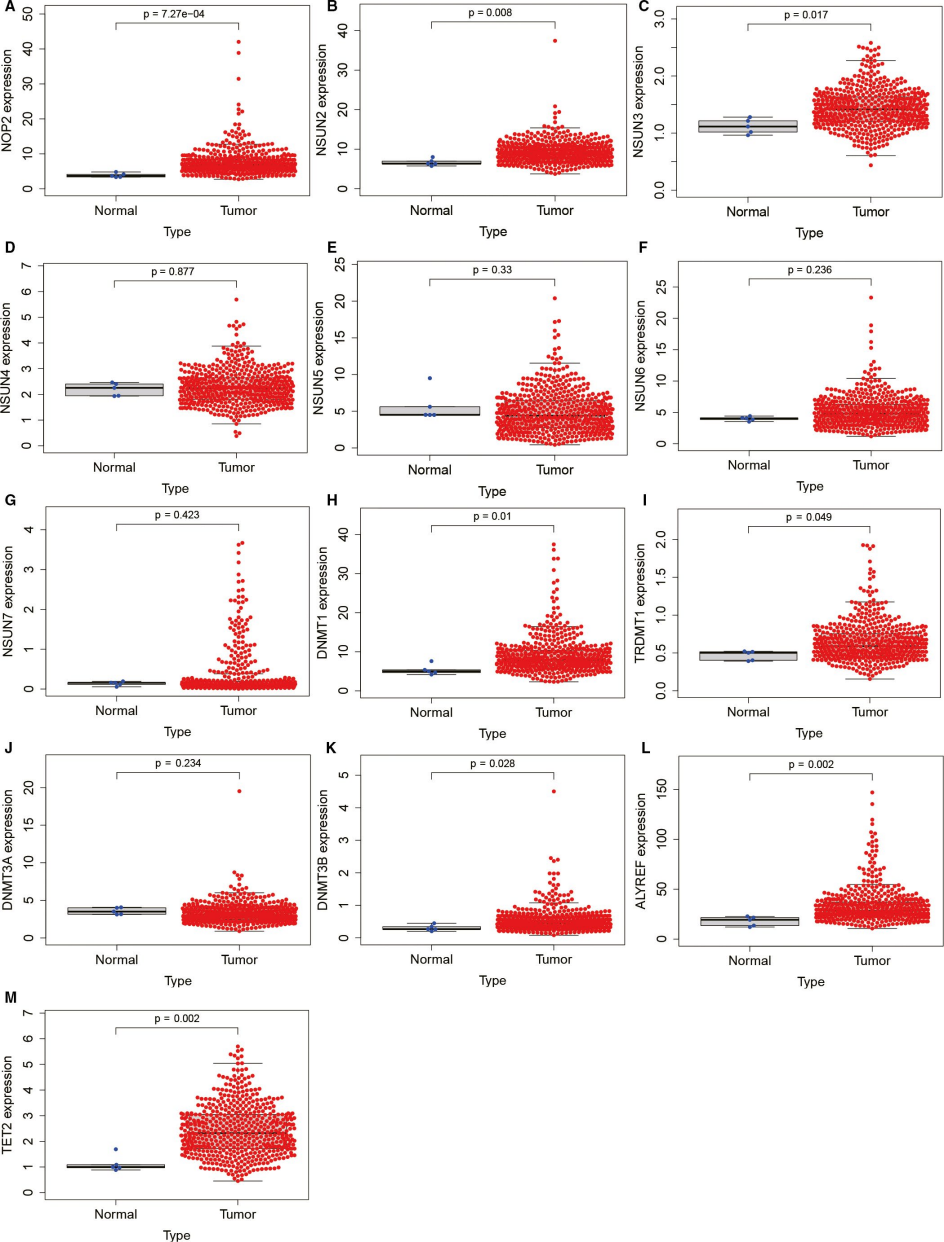
Expression of m^5^C regulators between low‐grade glioma and normal brain tissue

The basic characteristics of the patients of TCGA and CGGA datasets were shown in Table S1. We analysed the differentially expressed m^5^C regulators of the TCGA dataset and drew a heatmap to display the expression of m^5^C regulators in each low‐grade glioma sample, as shown in Figure 2. Eight differentially expressed m^5^C regulators (NSUN3, TET2, TRDMT1, ALYREF, DNMT3B, DNMT1, NOP2 and NSUN2) were identified. Interestingly, compared with normal brain tissue, these differentially expressed molecules were all up‐regulated in low‐grade glioma. The expressions of all m^5^C regulators between normal and tumour samples were shown in Figure 3.

**FIGURE 4:**
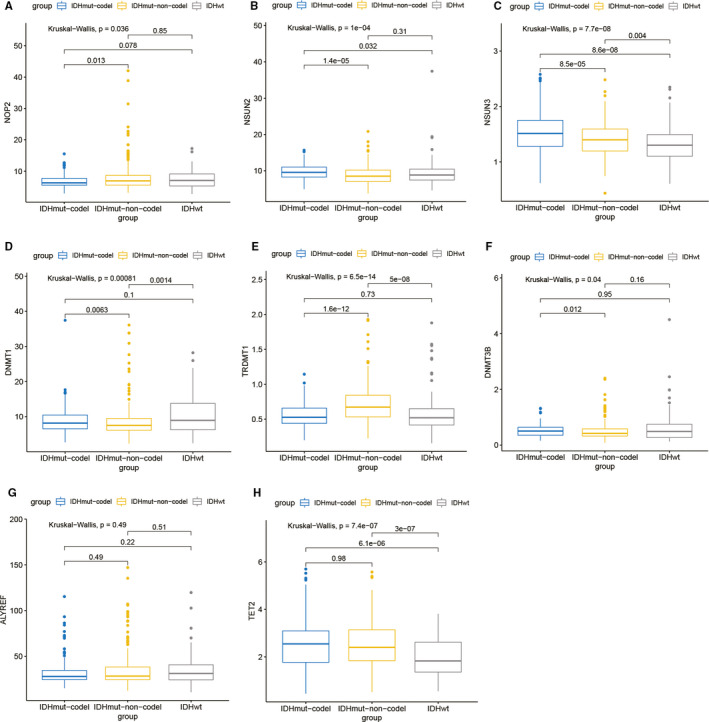
Expression of eight m^5^C regulators in different IDH and 1p/19q co‐deletion subtypes of low‐grade glioma (A: NOP2; B: NSUN2; C: NSUN3; D: DNMT1; E: TRDMT1; F: DNMT3B; G: ALYREF; H: TET2). Gene expression levels of 8 differentially expressed m^5^C regulators except ALYREF were diverse in different IDH and 1p/19q co‐deletion subtypes

Since different IDH and 1p/19q co‐deletion status has an impact on the prognosis of low‐grade glioma patients (survival outcomes in the IDH wild‐type group were worse compared with IDH mutant‐type group^13^ and survival outcomes in 1p/19q non‐co‐deletion group were worse compared with 1p/19q co‐deletion group^14^, ^15^), we evaluated the expression of these 8 differentially expressed genes in different IDH and 1p/19q co‐deletion status subgroups. The expression difference of TRDMT1 in each subtype was the most significant. TRDMT1 expression was higher in IDH wild‐type without 1p/19q co‐deletion group, while IDH wild‐type with 1p/19q co‐deletion group and IDH mutant group had lower TRDMT1 expression. Only ALYEF had no significant difference in expression in IDH and 1p/19q co‐deletion status subgroups. The detailed expression status of 8 differentially expressed molecules in each group was shown in Figure 4.

We also checked the expression of the 8 differentially expressed m^5^C regulators with different TERT promoter status, MGMT promoter status and ATRX status. Gene expression levels of NSUN2, NSUN3, DNMT1 and TRDMT1 were diverse in different TERT promoter status (Figure S1). In addition, gene expression levels of NSUN3 and TET2 were diverse in different MGMT promoter status (Figure S2). Furthermore, gene expression levels of NOP2, NSUN2, TRDMT1 and TET2 were diverse in different ATRX status (Figure S3).

### 3.3 Construction of a prognostic model based on m^5^C regulators

**FIGURE 5:**
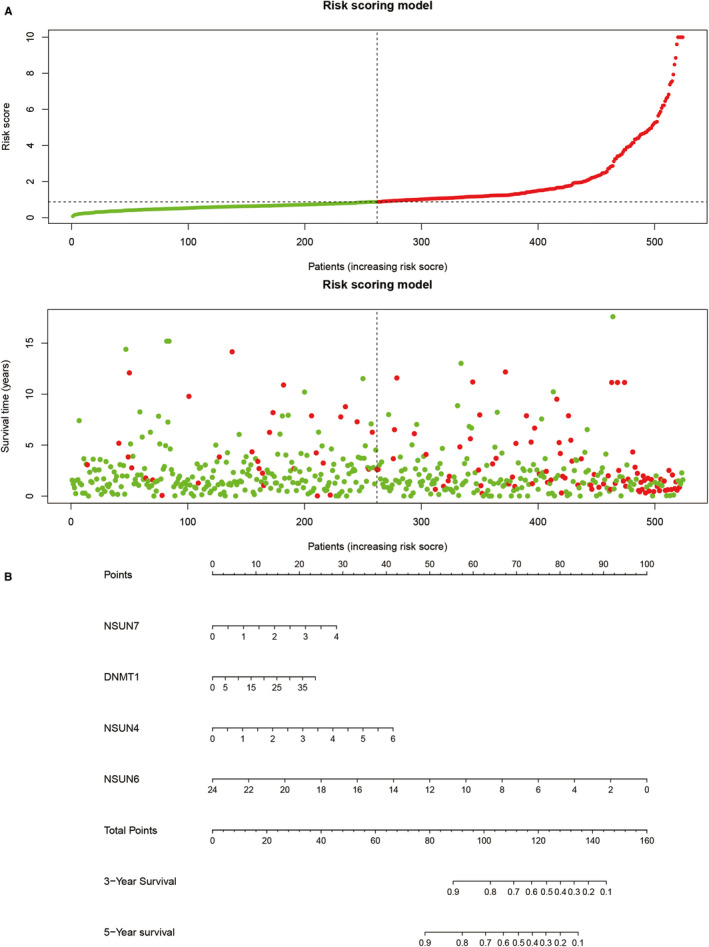
Prognostic model based on m^5^C regulators. (A) The correlation between overall survival and risk scores. (B) Nomogram of the prognostic model based on NSUN7, DNMT1, NSUN4 and NSUN6

At first, univariate Cox regression was applied to find prognosis‐related m^5^C regulators of low‐grade glioma. The results showed that NSUN4, NSUN7, DNMT1, DNMT3B, DNMT3A, NOP2 and NSUN5 were negatively correlated with the overall survival of low‐grade glioma, while NSUN6 was positively correlated with the overall survival. Based on multivariate Cox regression, a low‐grade glioma prognostic model was constructed. The risk score was expressed as: 0.594 * Expression_NSUN7_ + 0.369 * Expression_DNMT1_ + 0.892 * Expression_NSUN4_ – 1.055 * Expression_NSUN6_. The results of univariate and multivariate Cox analysis were shown in Table 1. The risk score and survival status of each sample were shown in Figure 5A. Meanwhile, Figure 5B presented the nomogram of the prognostic model for low‐grade glioma.

**TABLE 1:** The Cox regression results of the m^5^C regulators

RNA	Univariate Analysis		Multivariate Analysis	
	HR (95%CI)	*P*	HR (95%CI)	*P*
NSUN7	2.702 (2.054‐3.555)	0.000	1.812 (1.315‐2.496)	0.000
DNMT1	1.866 (1.369‐2.544)	0.000	1.446 (1.038‐2.014)	0.029
NSUN4	3.572 (1.830‐6.973)	0.000	2.439 (1.183‐5.030)	0.016
NSUN6	0.281 (0.184‐0.428)	0.000	0.348 (0.226‐0.536)	0.000

**FIGURE 6:**
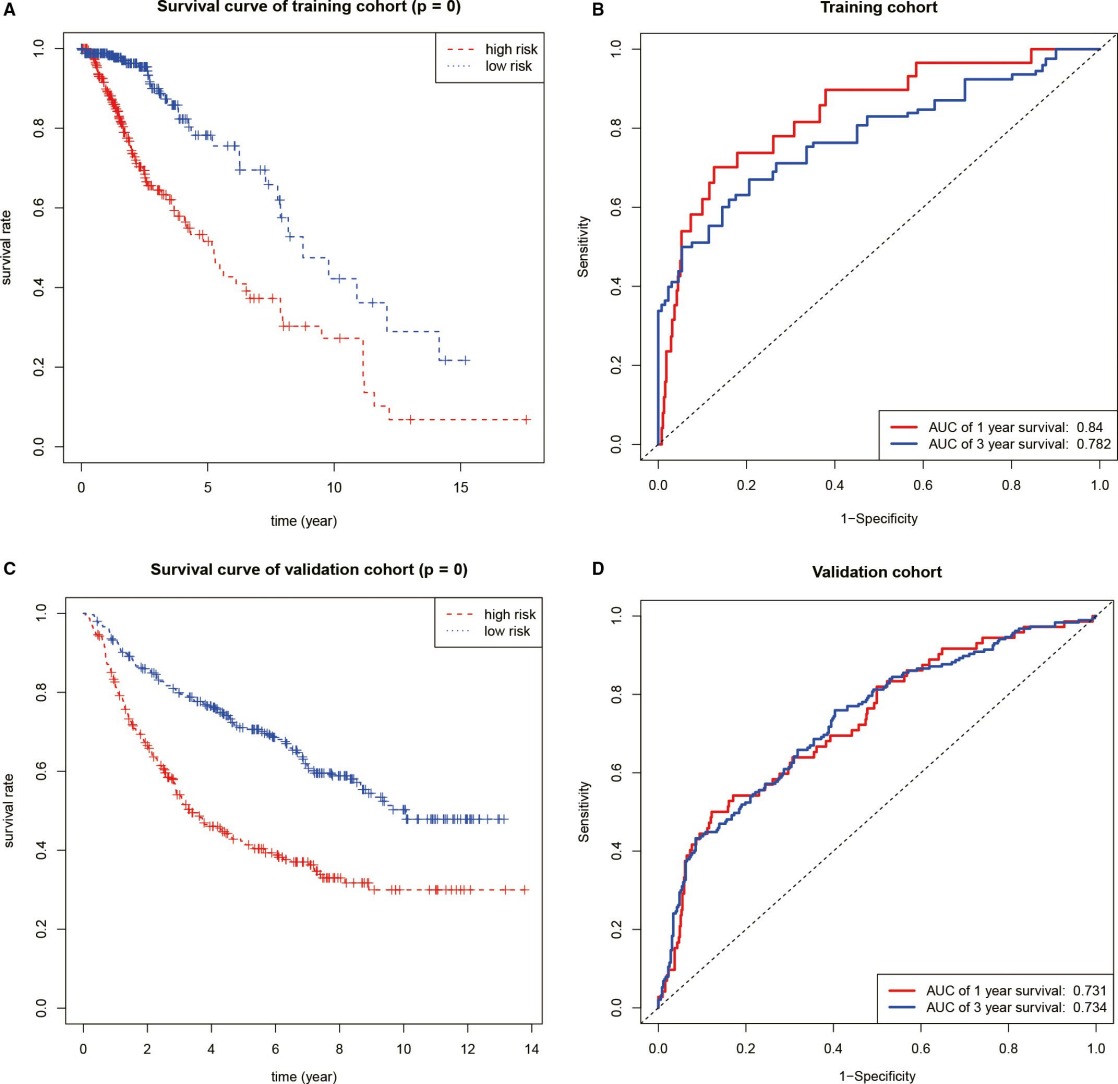
Testing of the prognostic model. (A) The prognosis of patients in low‐risk group of training cohort was remarkably better high‐risk group. (B) The ROC curves of the training cohort. (C) The prognosis of patients in low‐risk group of validation cohort was remarkably better than high‐risk group. (D) The ROC curves of the validation cohort

In order to verify the prognostic ability of the model for the overall survival of low‐grade glioma, Kaplan‐Meier curve and ROC curve were constructed, as shown in Figure 6. In training cohort, patients in low‐risk group had better prognosis compared with those in high‐risk group. (Figure 6A, *P* < .001). Besides, the area under the curve (AUC) of the 1‐year, 3‐year and 5‐year ROC curves was 0.84, 0.782 and 0.691, respectively (Figure 6B). In order to verify the repeatability of the model, this study used the CGGA dataset as the verification cohort for external verification. In the validation cohort, patients in low‐risk group also had better prognosis compared with those in high‐risk group. (Figure 6C, *P* < .001). And the AUC of the 1‐year, 3‐year and 5‐year ROC curves were 0.731, 0.734 and 0.717, respectively (Figure 6D).

### 3.4 GSEA

**FIGURE 7:**
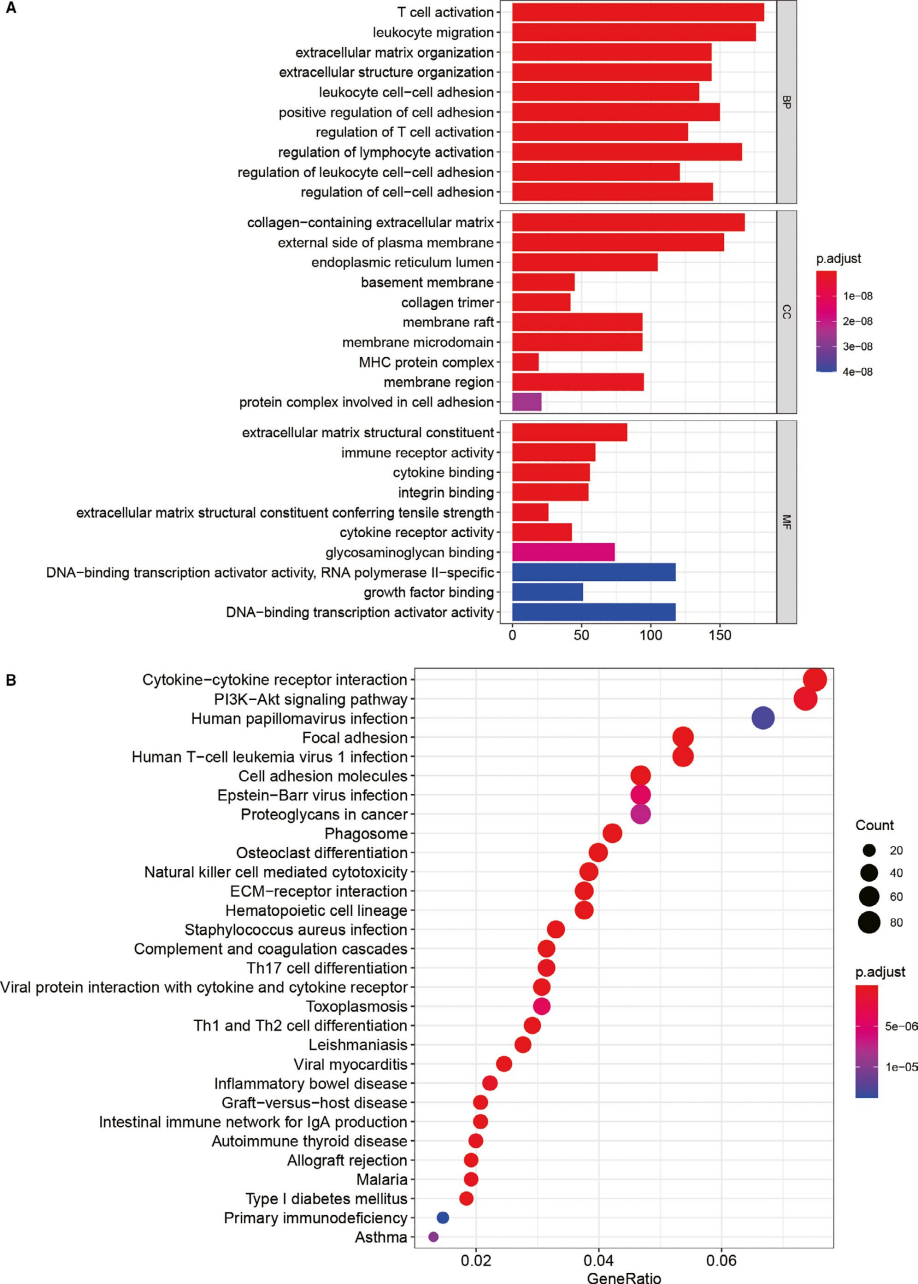
Functional enrichment analysis of m^5^C regulators. (A) GO enrichment results showed that m^5^C regulator‐related factors were enriched in DNA replication, chromosome segregation, nuclear division, etc (B) KEGG enrichment results showed that m^5^C regulator‐related factors were enriched in items such as cell cycle, P53 signalling pathway

In order to explore the possible functions of m^5^C regulators of low‐grade glioma, we performed GSEA on the m^5^C regulator‐related factors, as shown in Figure 7. Gene ontology (GO) enrichment analysis showed that m^5^C regulator‐related factors were enriched in DNA replication, chromosome segregation, nuclear division, etc Kyoto Encyclopedia of Genes and Genomes (KEGG) enrichment analysis showed that m^5^C regulator‐related factors were enriched in items such as cell cycle, P53 signalling pathway.

## 4 DISCUSSION

This study analysed the mutation and expression characteristics of m^5^C regulators in low‐grade glioma, and then analysed the relationship between these molecules and the prognosis and constructed a prognostic model. Kaplan‐Meier curve and ROC curve were used in both training cohort and validation cohort to test the predictive ability of prognostic model of low‐grade glioma. Finally, GSEA was used to predict the biological functions of m^5^C regulators in low‐grade glioma.

m^5^C methylation is widespread in DNA and RNA modification.^10^, ^16^ Its principal process includes the catalysis of cytosine at position 5 by the ‘writers’ and then be recognized by the ‘reader’. m^5^C methylation mainly occurs in the vicinity of the start codon and the 3`UTR region of mRNAs in the transcriptome.^11^, ^17^ Although m^5^C methylation has been proved to be widespread in eukaryotic cells,^12^, ^18^ the relationship between m^5^C methylation and glioma is very limited. Since gene mutation may be the driving factor of expression, this study first studied the variation of m^5^C regulators. We found that DNMT3A mutation was the most frequent. However, the overall frequency of these m^5^C regulators in all low‐grade glioma was not high (15/506).

Next, we studied the expression of these m^5^C regulators. The results showed that NSUN3, TET2, TRDMT1, ALYREF, DNMT3B, DNMT1, NOP2 and NSUN2 were up‐regulated in low‐grade glioma tissues, some of which have been studied in tumours. For example, knockdown of TRDMT1 can change the level of HEK293 mRNA methylation and inhibit tumour cell proliferation and migration.^19^ In addition, there have been reports that NSUN2 is significantly up‐regulated in gastric cancer, inhibits the expression of CDKN1C in an m^5^C‐dependent manner and promotes the proliferation of gastric cancer cells.^20^ In human urothelial carcinoma of bladder, NSUN2 targets and regulates HDGF through m^5^C methylation, thereby promoting tumorigenesis.^21^ Overall, m^5^C regulators have been reportedly limited in tumours, and even less in low‐grade glioma. Further, we also studied the expression of these differentially expressed m^5^C regulators with different IDH status, 1p/19q co‐deletion status, TERT promoter status, MGMT promoter status and ATRX status. However, our results showed these m^5^C regulators had various expression levels in different subgroups. The mechanisms by which these m^5^C regulators regulate different subgroups of glioma deserve more in‐depth study.

Through survival analysis, this study constructed a low‐grade glioma prognostic model with 4 genes (NSUN4, NSUN7, DNMT1 and NSUN6) and drew a nomogram accordingly. *P* value of Kaplan‐Meier curve of CGGA validation cohort was quite significant. Besides, the AUC values were 0.731, 0.734 and 0.717 in 1‐year, 3‐year and 5‐year ROC curves in CGGA validation cohort. The prognostic model had a strong predictive power for the overall survival for low‐grade glioma patients. Overall, the m^5^C regulator signatures showed great value for clinical applications in low‐grade glioma.

This study conducted enrichment analysis of m^5^C regulator‐related factors, and the results showed that these factors were enriched in items such as DNA replication, chromosome segregation, nuclear division, cell cycle and P53 signalling pathway. It is reported that up‐regulated LINC00467 can bind to DNMT1 and inhibit p53 level and promote glioma cells proliferation and invasion.^22^ Furthermore, lncRNA SNHG7 regulates p53 expression and arrests cell cycle in the G0/G1 phase of osteosarcoma by binding with DNMT1.^23^ These indicated that m^5^C regulators might have close relationship with tumour cell proliferation and are of significance for glioma research.

This study still has some limitations. First, results obtained based on sequencing datasets still need to be examined by basic experiments. Second, more potential m^5^C regulators have yet to be discovered.

We studied the expression and prognostic characteristics of m^5^C regulators in low‐grade glioma, supplied biomarkers for the diagnosis and prognosis and provided the foundation for the study of the pathogenesis of low‐grade glioma.

## CONFLICT OF INTEREST

None declared.

## AUTHOR CONTRIBUTIONS

**Xiaozhi Li:** Conceptualization (equal); Data curation (lead); Formal analysis (lead); Funding acquisition (lead); Investigation (supporting); Methodology (lead); Project administration (lead); Resources (lead); Software (lead); Supervision (supporting); Validation (lead); Visualization (equal); Writing‐original draft (lead); Writing‐review & editing (lead). **Yutong Meng:** Conceptualization (equal); Data curation (supporting); Formal analysis (supporting); Funding acquisition (supporting); Investigation (lead); Methodology (supporting); Project administration (supporting); Resources (supporting); Software (supporting); Supervision (lead); Validation (supporting); Visualization (equal); Writing‐original draft (supporting); Writing‐review & editing (supporting).

## ETHICAL APPROVAL

No more ethical approval was needed since all data of this study were acquired from public database.

## Supporting information

Figure S1

Figure S2

Figure S3

Table S1

## Data Availability

Data were available from corresponding author on reasonable request.
