# Supplementary figures and images for: Expression and prognostic characteristics of m5C regulators in low‐grade glioma
Source: J Cell Mol Med. 2021 Jan 5;25(3):1383–93. doi: 10.1111/jcmm.16221 (PMC7875931; doi:10.1111/jcmm.16221)

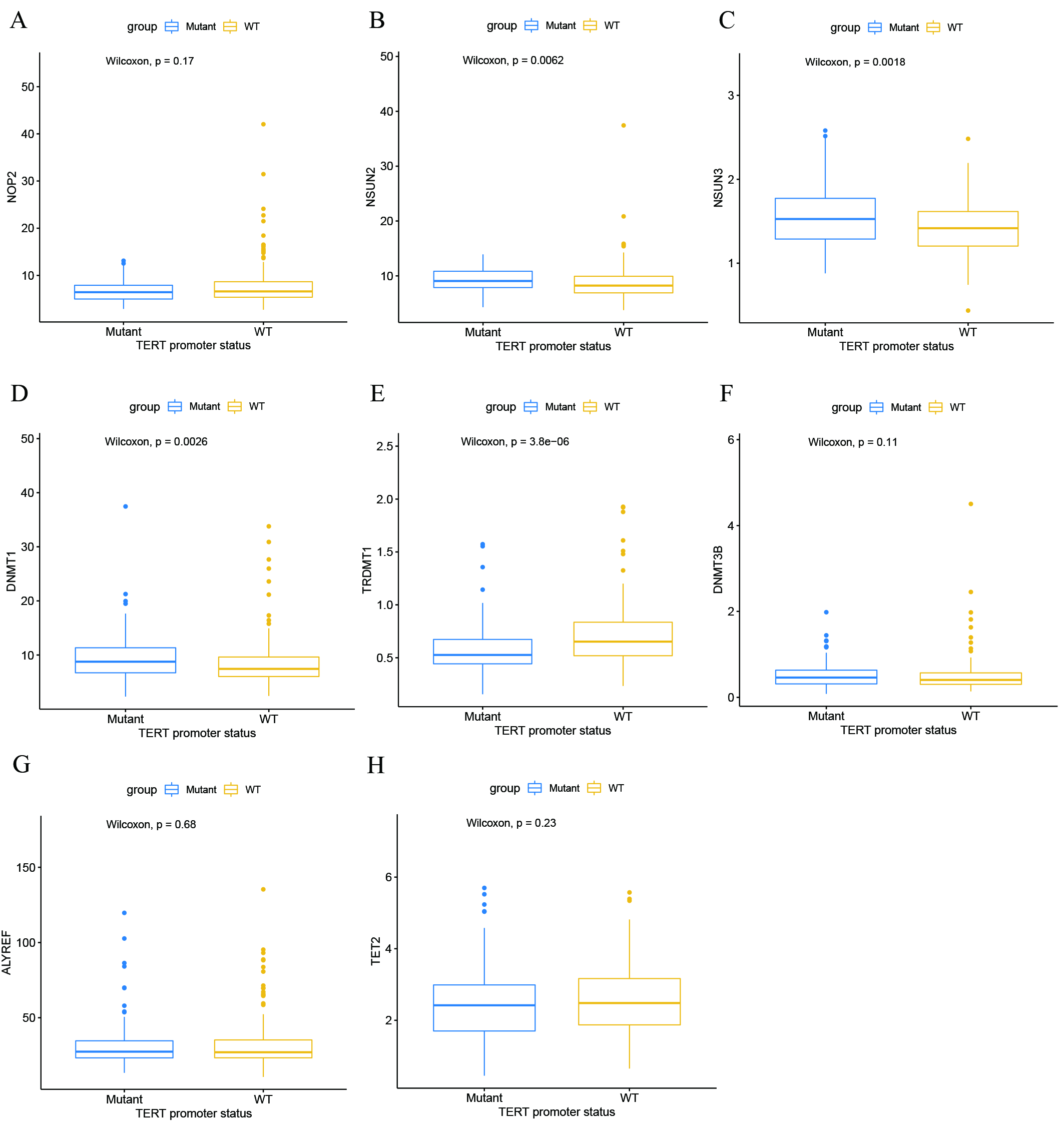

Supplement: Supplementary file 1 — Figure S1 [file JCMM-25-1383-s001.tif]

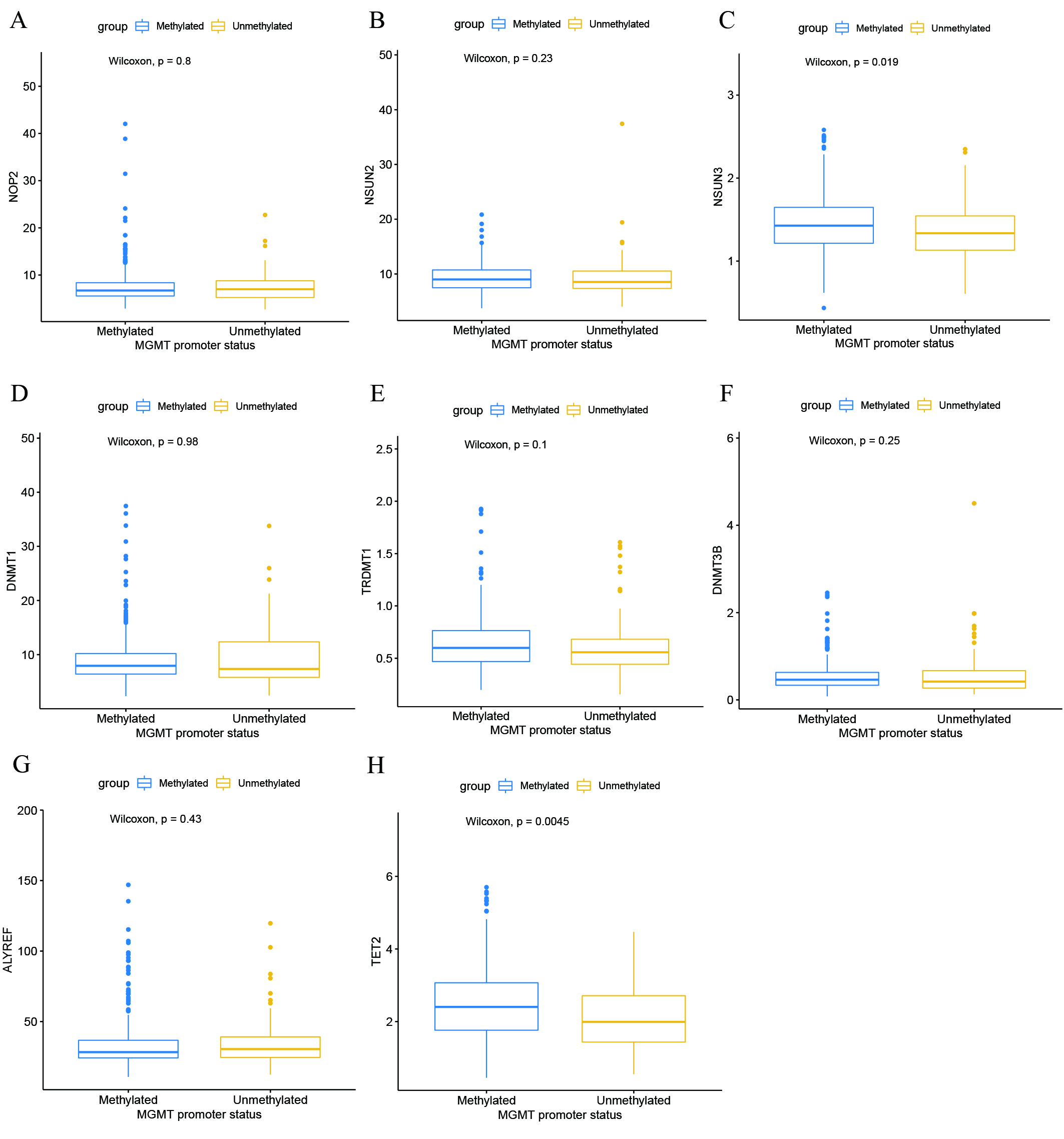

Supplement: Supplementary file 2 — Figure S2 [file JCMM-25-1383-s002.tif]

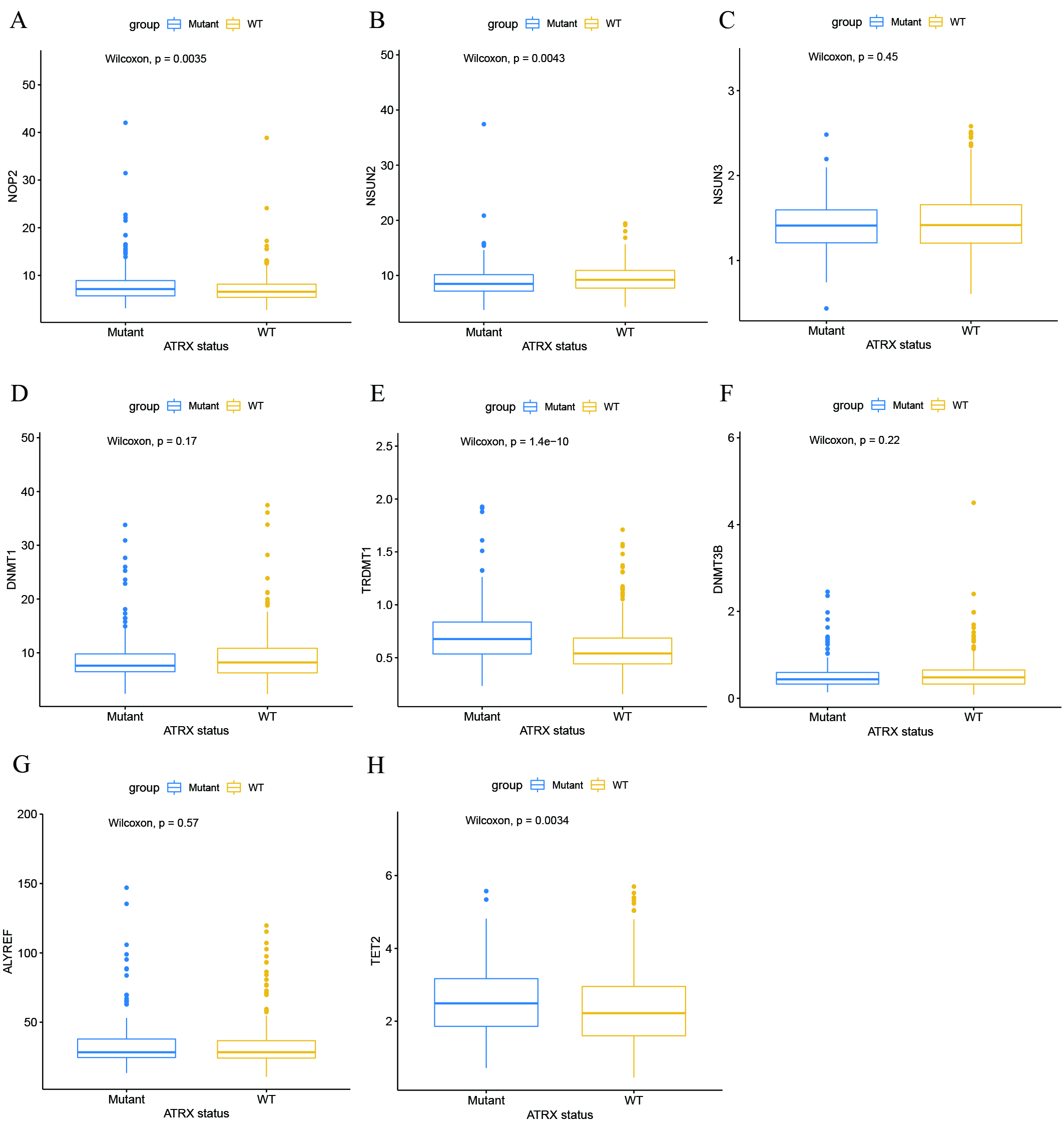

Supplement: Supplementary file 3 — Figure S3 [file JCMM-25-1383-s003.tif]
