# Supplementary material for: Expression and prognostic characteristics of m5C regulators in low‐grade glioma
Source: J Cell Mol Med. 2021 Jan 5;25(3):1383–93. doi: 10.1111/jcmm.16221 (PMC7875931; doi:10.1111/jcmm.16221)
Supplement: Supplementary file 4 — Table S1 [file JCMM-25-1383-s004.doc]

| Table S1. Patient characteristics of TCGA and CGGA datasets | | | |
| --- | --- | --- | --- |
|  |  | **TCGA dataset** | **CGGA dataset** |
| **Cases** |  | 506 | 592 |
| **Age** | Age ≤ 30 | 96 | 103 |
|  | 30 ＞ Age ≤ 40 | 153 | 206 |
|  | 40 ＞ Age ≤ 50 | 103 | 192 |
|  | Age ＞ 50 | 154 | 94 |
| **Gender** | Female | 226 | 251 |
|  | Male | 280 | 341 |
| **Overall survival** | Dead | 117 | 287 |
|  | Alive | 389 | 305 |
